# Supplementary material for: Noradrenergic consolidation of social recognition memory is mediated by β-arrestin–biased signaling in the mouse prefrontal cortex
Source: Commun Biol. 2022 Oct 17;5:1097. doi: 10.1038/s42003-022-04051-y (PMC9576713; doi:10.1038/s42003-022-04051-y)
Supplement: Supplementary file 2 — Supplementary information [file 42003_2022_4051_MOESM2_ESM.pdf]

Supplementary Information

Noradrenergic consolidation of social recognition memory is mediated by β-arrestin–biased signaling in the mouse prefrontal cortex

Deqin Cheng[1,2], Junwen Wu[1,2], Enhui Yan[1,2], Xiaocen Fan[1,2], Feifei Wang[1,2], Lan Ma[1,2*], Xing Liu[1,2*]

[1]School of Basic Medical Sciences, State Key Laboratory of Medical Neurobiology, MOE Frontiers Center for Brain Science, Institutes of Brain Science, Department of Neurology, Pharmacology Research Center, Huashan Hospital, Fudan University, Shanghai 200032, China.

[2]Research Unit of Addiction Memory, Chinese Academy of Medical Sciences (2021RU009), Shanghai 200032, China.

* Corresponding author. Email: xingliu@fudan.edu.cn (X Liu); lanma@fudan.edu.cn (L Ma)

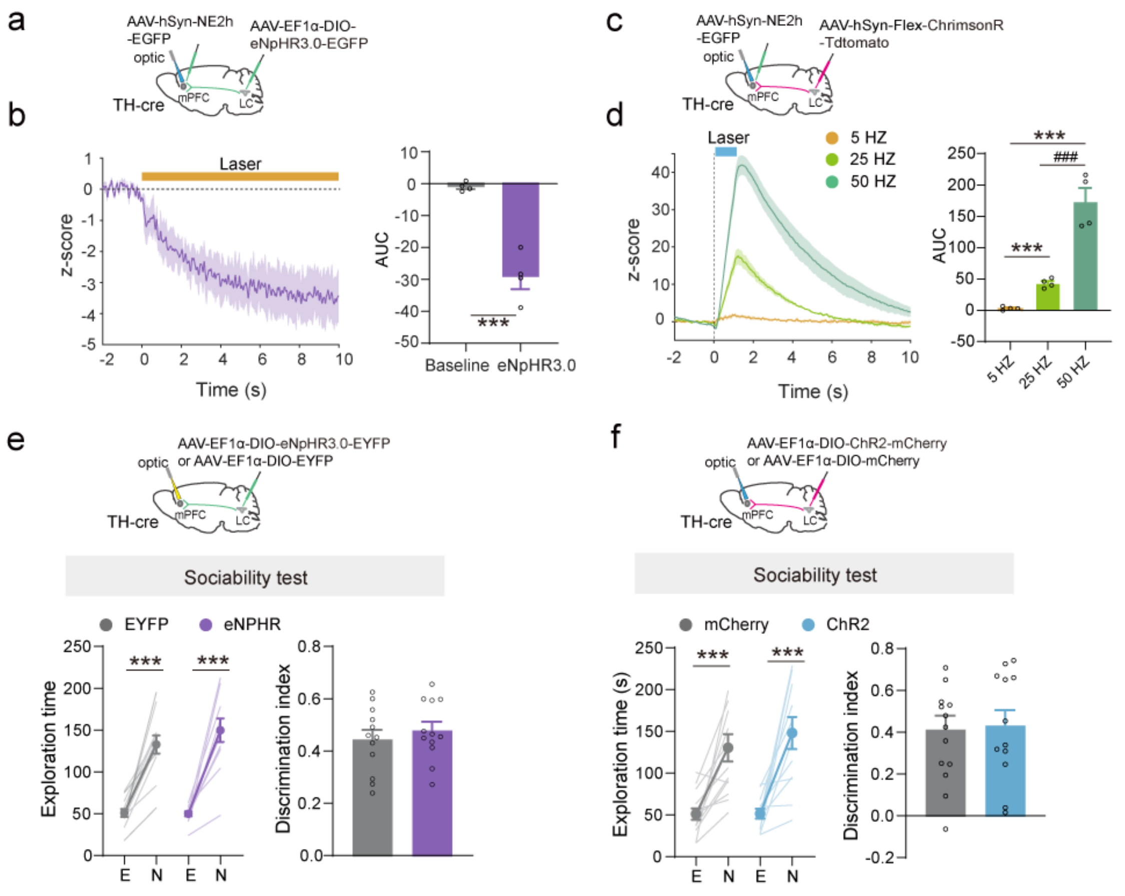

Supplementary Fig. 1. The sociability in laser stimulation and control groups.

a, c Photometry recording of NE release. *AAV9-EF1α-DIO-eNpHR3.0-EYFP* or *AAV9-EF1α-Flex-ChrimsonR-Tdtomato* was injected in the LC, *AAV9-hSyn-NE2h-EGFP* was injected in the mPFC of TH-Cre mice. Optical fibers were implanted above the mPFC. **b, d** Left: Plot of grouped average fluorescence in response to optical stimulation. Right: Bar graph of Mean Z-score for a 10-s window after the onset of optical stimulation. (**b**, eNpHR3.0: n = 4. **d**, ChrimosonR: n = 4). **e, f** Statistical graphs of exploration time for empty cage (E) and novel mouse (N) and discrimination scores in sociability test (**e**, n = 12 for each group. **f**, mCherry: n = 14, ChR2: n = 12). $^{***}p < 0.001$ and $^{###}p < 0.001$ vs indicated group.

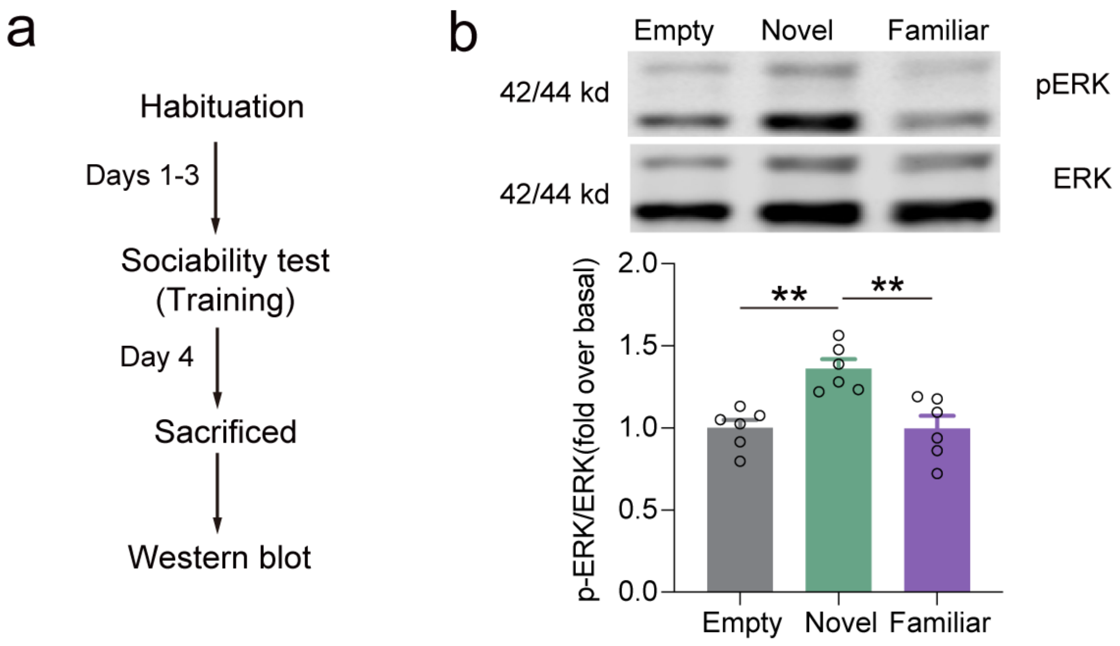

Supplementary Fig. 2. Novel mouse exposure increases the pERK levels in the mPFC.

a Experimental scheme. The experiment mice were group housed with juvenile mice for three days. During sociability tests, the mice were exposed to the three-chamber containing a wire cage with a familiar or a novel mouse. 15 min after sociability test, pERK levels in the mPFC were examined. **b** Representative western blots and bar graph for pERK levels in the mPFC after sociability test (n = 6 for each group). $^{**}p < 0.01$ vs indicated group.

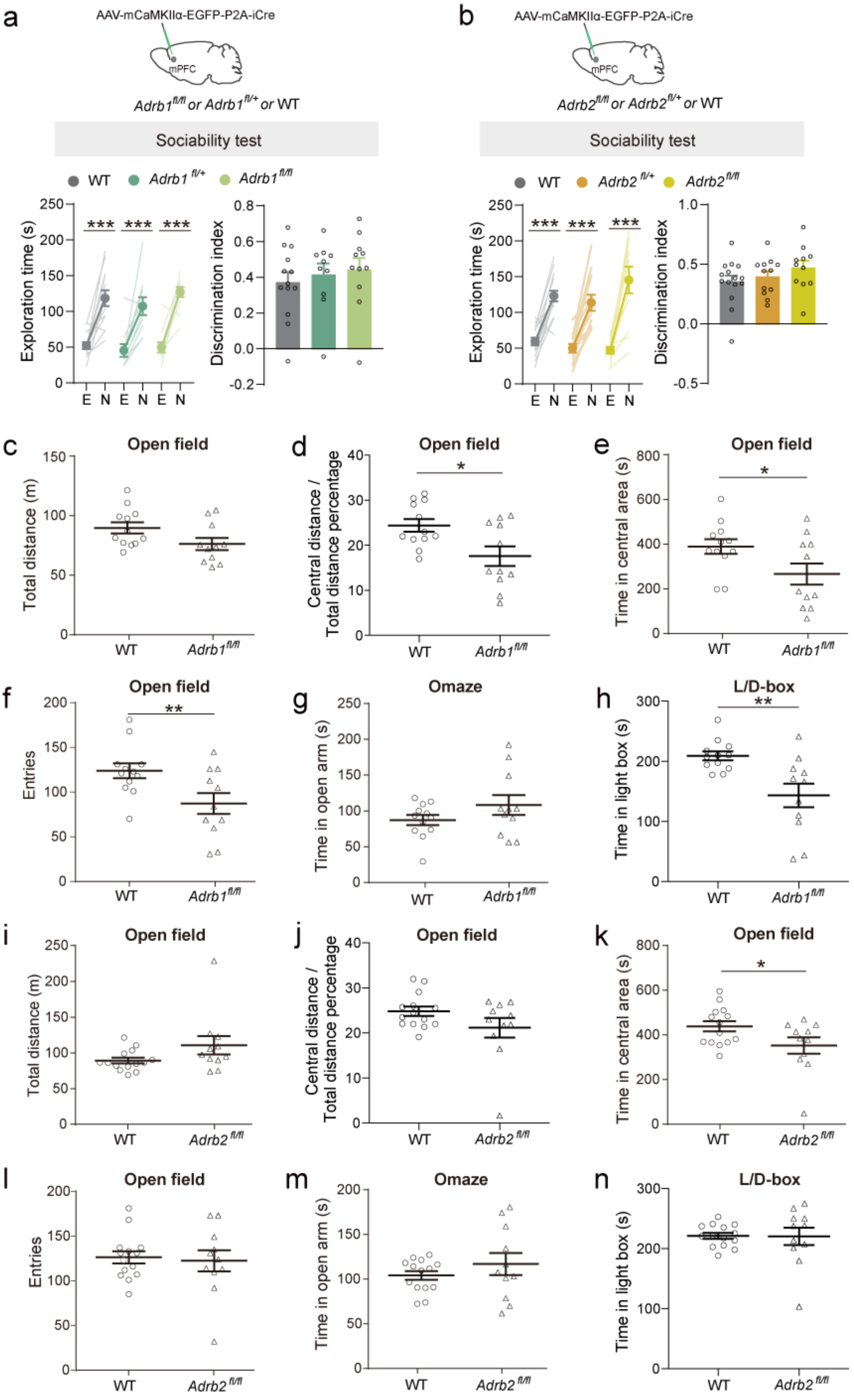
a
AAV-mCaMKIIα-EGFP-P2A-iCre
mPFC
Adrb1fl/fl or Adrb1fl/+ or WT
Sociability test
WT
Adrb1 fl/+
Adrb1 fl/fl
Exploration time (s)
Discrimination index
E N E N E N
b
AAV-mCaMKIIα-EGFP-P2A-iCre
mPFC
Adrb2fl/fl or Adrb2fl/+ or WT
Sociability test
WT
Adrb2 fl/+
Adrb2 fl/fl
Exploration time (s)
Discrimination index
E N E N E N
c
Open field
Total distance (m)
WT
Adrb1fl/fl
d
Open field
Central distance / Total distance percentage
WT
Adrb1fl/fl
e
Open field
Time in central area (s)
WT
Adrb1fl/fl
f
Open field
Entries
WT
Adrb1fl/fl
g
Omaze
Time in open arm (s)
WT
Adrb1fl/fl
h
L/D-box
Time in light box (s)
WT
Adrb1fl/fl
i
Open field
Total distance (m)
WT
Adrb2 fl/fl
j
Open field
Central distance / Total distance percentage
WT
Adrb2 fl/fl
k
Open field
Time in central area (s)
WT
Adrb2 fl/fl
l
Open field
Entries
WT
Adrb2 fl/fl
m
Omaze
Time in open arm (s)
WT
Adrb2 fl/fl
n
L/D-box
Time in light box (s)
WT
Adrb2 fl/fl

Supplementary Fig. 3. β1-AR or β2-AR knockout in the mPFC increases anxiety levels.

a, b Statistical graphs of exploration time for empty cage (E) and novel mouse (N) and discrimination scores in sociability test in the mice with β1-AR or β2-AR knockout in the mPFC (**a**, WT: n = 13, *Adrb1*$^{fl/+}$: n = 10, *Adrb1*$^{fl/fl}$: n = 11. **b**, WT: n = 15, *Adrb2*$^{fl/+}$: n = 12, *Adrb2*$^{fl/fl}$: n = 11). **c-n** Open filed test, O-maze test and L/D-box test in *Adrb1*$^{fl/fl}$, *Adrb2*$^{fl/fl}$ and their WT littermates. WT: n = 12, *Adrb1*$^{fl/fl}$: n = 11; WT: n = 14, *Adrb2*$^{fl/fl}$: n = 11. **c-f, i-l** The open filed test. **c, i** The total distance in the open filed. **d, j** Percentage of central distance/ total distance. **e, k** Duration in the central area in open filed test. **f, l** The entries to the central area. **g, m** Duration in the open arm of *Adrb1*$^{fl/fl}$, *Adrb2*$^{fl/fl}$ and their WT littermates in O-maze test. **h, n** Duration in the light box of *Adrb1*$^{fl/fl}$, *Adrb2*$^{fl/fl}$ and their WT littermates in L/D-box test. $^{*}p < 0.05$, $^{**}p < 0.01$ and $^{***}p < 0.001$ vs indicated group.

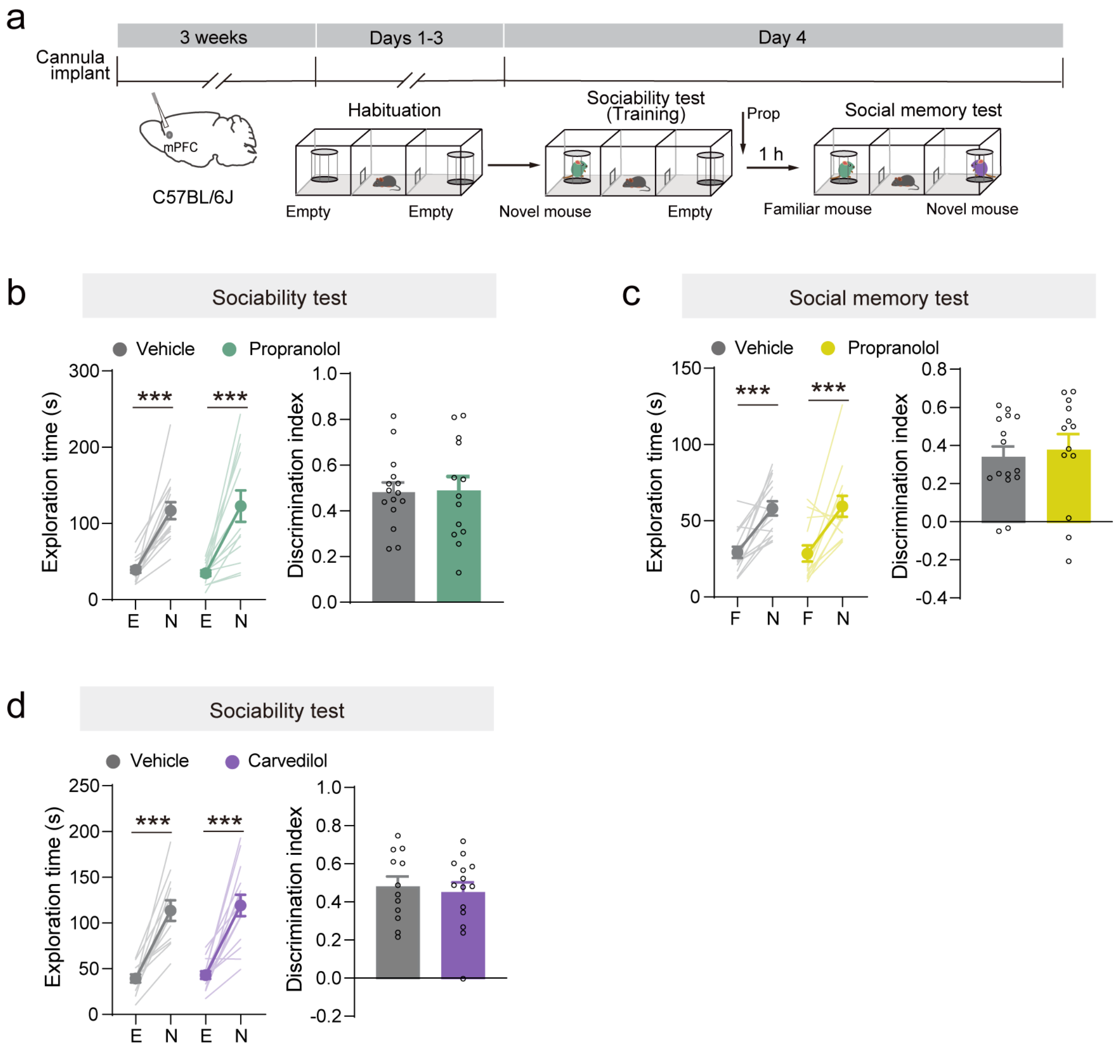

Supplementary Fig. 4. Propranolol infusion in the mPFC dose not impair short-term memory of SRM.

a Experimental scheme. Propranolol (Prop, 10 μg) was infused in the mPFC after sociability test and social memory tests were carried out 1 h later. **b, d** Statistical graphs of exploration time for empty cage (E) and novel mouse (N) and discrimination scores in sociability test (**b**, Vehicle: n = 15, Propranolol: n = 13. **d**, Vehicle: n = 12, Carvedilol: n = 14). **c** Statistical graphs of exploration time and discrimination scores in social memory test. (Vehicle: n = 15, Propranolol: n = 13). $^{***}p < 0.001$ vs indicated group.

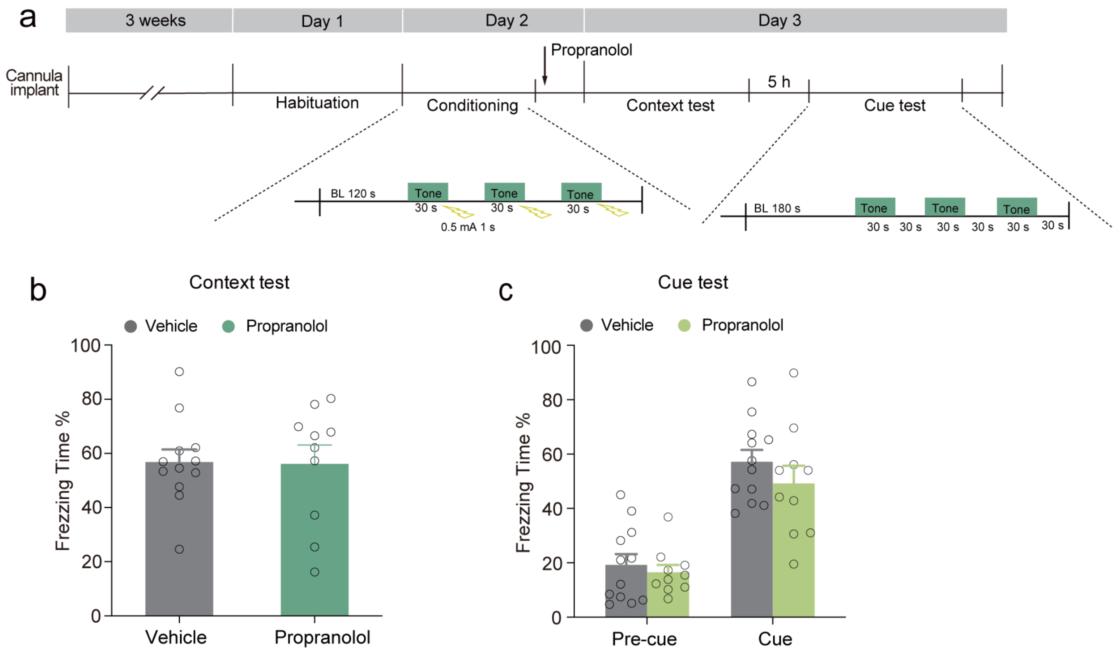

Supplementary Fig. 5. Propranolol infusion in the mPFC dose not impair fear memory consolidation.

a Experimental scheme. Propranolol (Prop, 10 μg) was infused in the mPFC after fear conditioning. Contextual or cue fear memory tests were carried out 1 day later. **b, c** Statistical graphs of freezing levels (Vehicle: n = 12, Propranolol: n = 10).

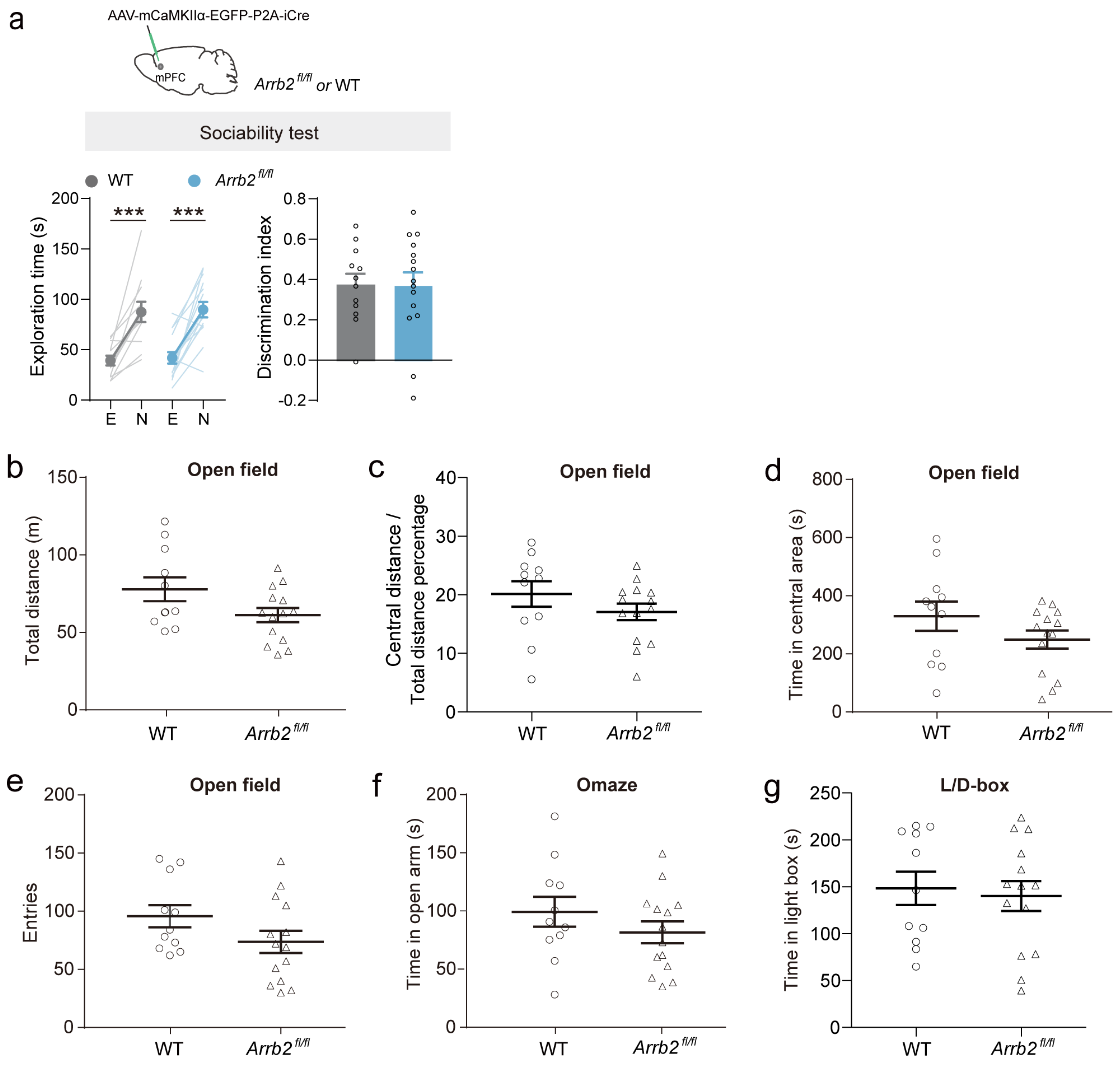

Supplementary Fig. 6. β-arrestin2 knockout in the mPFC does not significantly change anxiety levels.

a Statistical graphs of exploration time for empty cage (E) and novel mouse (N) and discrimination scores in sociability test in the mice with β-arretin2 knockout in the mPFC. (WT: n = 12, *Arrb2*$^{fl/fl}$: n = 15). **b-g** Open field test, O-maze test and L/D-box test in *Arrb2*$^{fl/fl}$ and their WT littermates. WT: n = 11, *Arrb2*$^{fl/fl}$: n = 14. **b-e** The open field test. **b** The total distance in open filed. **c** Percentage of central distance/total distance. **d** Duration in the central area. **e** The entries to the central area. **f** Duration in the open arm of *Arrb2*$^{fl/fl}$ and their WT littermates in O-maze test. **g** Duration in the light box of *Arrb2*$^{fl/fl}$ and their WT littermates in L/D-box test. $^{***}p < 0.001$ vs indicated group.

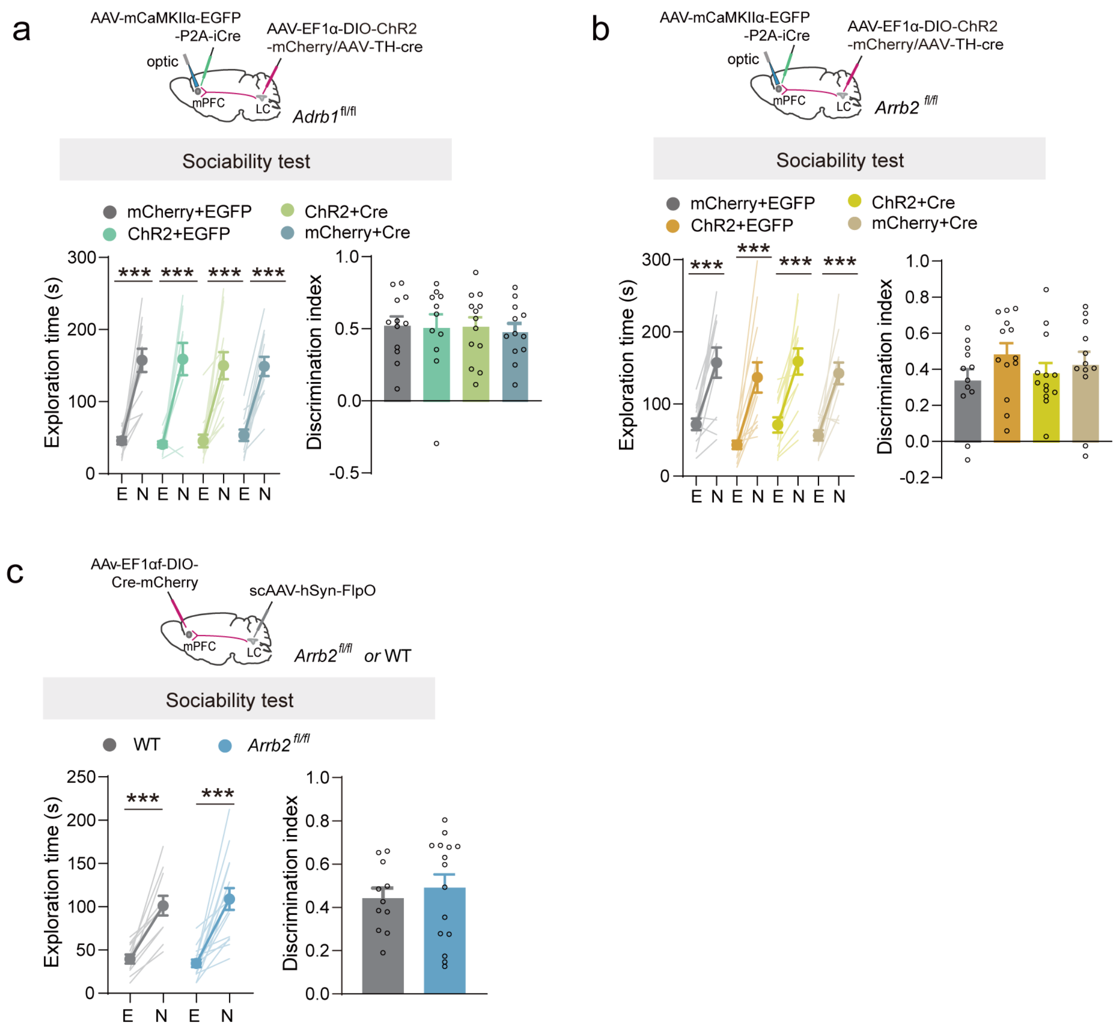

Supplementary Fig. 7. The sociability in laser stimulation and control groups combined with β-arrestin2 knockout in the LC-mPFC circuits.

a-c Statistical graphs of exploration time for empty cage (E) and novel mouse (N) and discrimination scores in sociability test. (**a**, mCherry/EGFP, n =12, ChR2/EGFP, n = 11, ChR2/Cre, n = 13, mCherry/Cre, n = 12. **b**, mCherry/EGFP, n = 12; ChR2/EGFP, n = 13; ChR2/Cre, n = 13; mCherry/Cre, n = 12. **c**, WT: n = 11; *Arrb2$^{fl/fl}$*: n = 15). $^{***}p < 0.001$ vs indicated group.

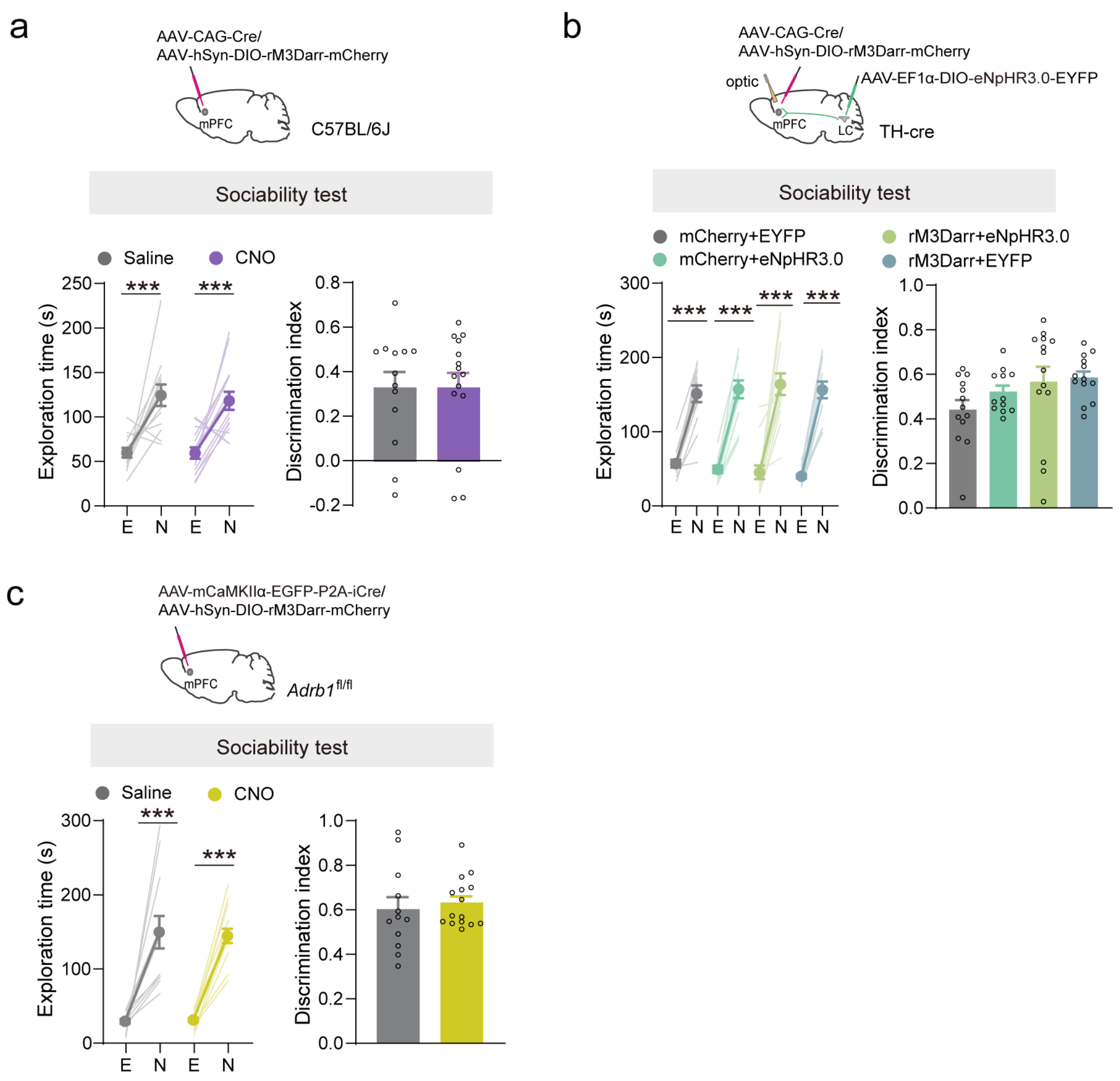

Supplementary Fig. 8. The sociability in laser stimulation and control groups combined with β-arrestin2 signaling activation.

a-c Statistical graphs of exploration time for empty cage (E) and novel mouse (N) and discrimination scores in sociability test. (**a**, Saline, n = 13; CNO, n = 15. **b**, EYFP/mCherry, n = 13; EYFP/rM3Darr, n = 12; eNpHR3.0/rM3Darr, n = 14; eNpHR3.0/mCherry, n = 13. **c**, Saline, n = 12; CNO, n = 15). $^{***}p < 0.001$ vs indicated group.

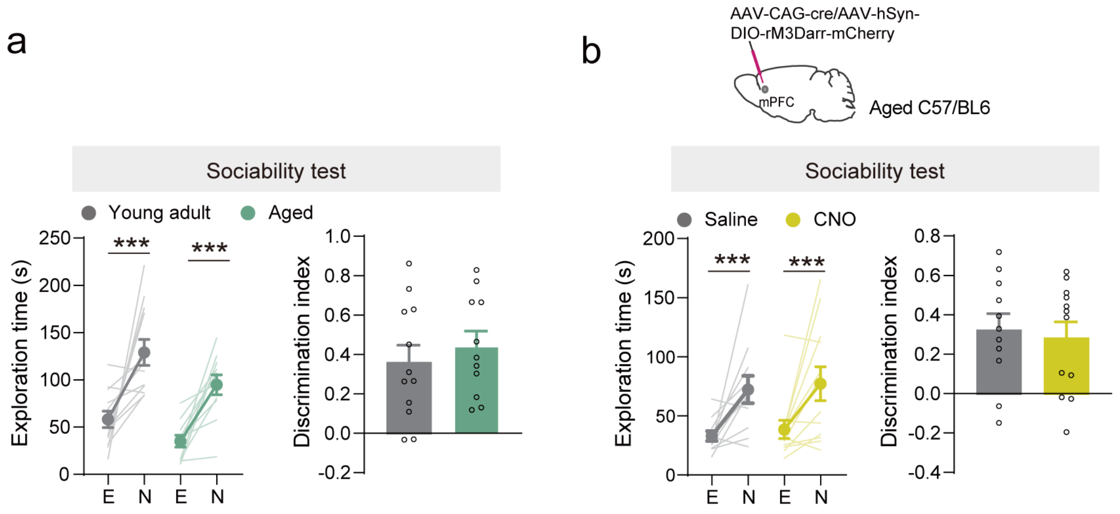

Supplementary Fig. 9. The sociability is intact in aged mice.

a, b Statistical graphs of exploration time for empty cage (E) and novel mouse (N) and discrimination scores in sociability test (**a**. Young adult, n = 12; Aged, n = 11. **b**, Saline, n = 11; CNO, n = 12). $^{***}p < 0.001$ vs indicated group.

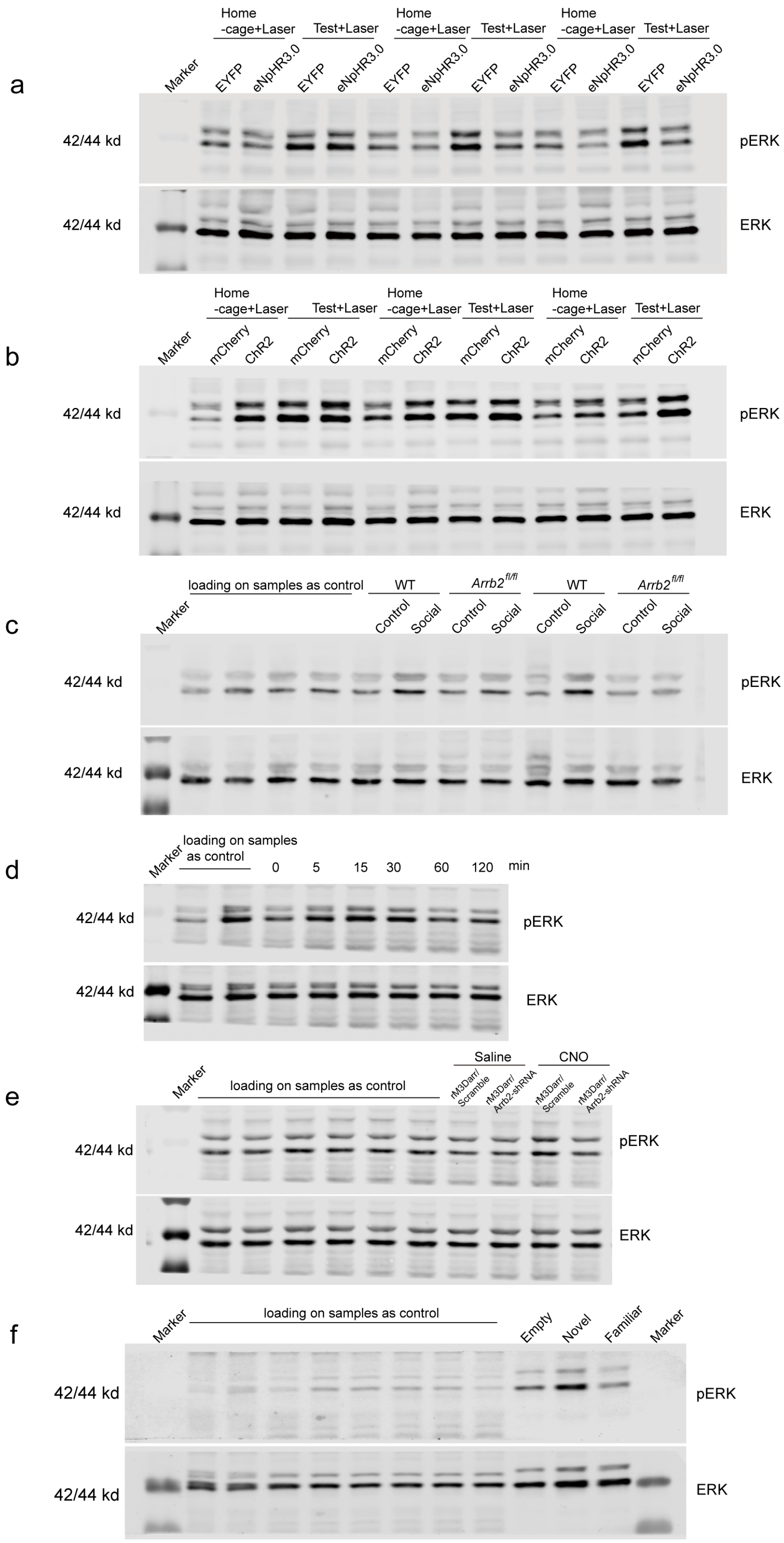
a
Marker
Home -cage+Laser
Test+Laser
EYFP
eNpHR3.0
42/44 kd
pERK
ERK
b
Marker
Home -cage+Laser
Test+Laser
mCherry
ChR2
42/44 kd
pERK
ERK
c
Marker
loading on samples as control
WT
Arrb2 fl/fl
Control
Social
42/44 kd
pERK
ERK
d
Marker
loading on samples as control
0
5
15
30
60
120
min
42/44 kd
pERK
ERK
e
Marker
loading on samples as control
Saline
CNO
rM3Darr/ Scramble
rM3Darr/ Arrb2-shRNA
42/44 kd
pERK
ERK
f
Marker
loading on samples as control
Empty
Novel
Familiar
42/44 kd
pERK
ERK

Supplementary Fig. 10. The uncropped blots image.

a The uncropped versions of Fig.1n blots. **b** The uncropped versions of Fig.1o blots. **c** The uncropped versions of Fig.3g blots. **d** The uncropped versions of Fig.5a blots. **e** The uncropped versions of Fig.5b blots. **f** The uncropped versions of Supplementary Fig.2b blots.
