# Supplementary material for: Noradrenergic consolidation of social recognition memory is mediated by β-arrestin–biased signaling in the mouse prefrontal cortex
Source: Commun Biol. 2022 Oct 17;5:1097. doi: 10.1038/s42003-022-04051-y (PMC9576713; doi:10.1038/s42003-022-04051-y)
Supplement: Supplementary file 3 — Description of Additional Supplementary Data [file 42003_2022_4051_MOESM3_ESM.docx]

**Description of Additional Supplementary Files**

**File name:** Supplementary Data 1

**Description:** Statistical detail information for figures

**File name:** Supplementary Data 2

**Description:** The source data behind the graphs in the paper
