# Supplementary material for: Noradrenergic consolidation of social recognition memory is mediated by β-arrestin–biased signaling in the mouse prefrontal cortex
Source: Commun Biol. 2022 Oct 17;5:1097. doi: 10.1038/s42003-022-04051-y (PMC9576713; doi:10.1038/s42003-022-04051-y)
Supplement: Supplementary file 6 — Reporting Summary [file 42003_2022_4051_MOESM6_ESM.pdf]

nature portfolio

Corresponding author(s): Xing Liu, Lan Ma

Last updated by author(s): Sep 23, 2022

Reporting Summary

Nature Portfolio wishes to improve the reproducibility of the work that we publish. This form provides structure for consistency and transparency in reporting. For further information on Nature Portfolio policies, see our Editorial Policies and the Editorial Policy Checklist.

Statistics

For all statistical analyses, confirm that the following items are present in the figure legend, table legend, main text, or Methods section.

n/a	Confirmed	
☐	☒	The exact sample size (*n*) for each experimental group/condition, given as a discrete number and unit of measurement
☐	☒	A statement on whether measurements were taken from distinct samples or whether the same sample was measured repeatedly
☐	☒	The statistical test(s) used AND whether they are one- or two-sided<br>*Only common tests should be described solely by name; describe more complex techniques in the Methods section.*
☐	☒	A description of all covariates tested
☐	☒	A description of any assumptions or corrections, such as tests of normality and adjustment for multiple comparisons
☐	☒	A full description of the statistical parameters including central tendency (e.g. means) or other basic estimates (e.g. regression coefficient) AND variation (e.g. standard deviation) or associated estimates of uncertainty (e.g. confidence intervals)
☐	☒	For null hypothesis testing, the test statistic (e.g. *F*, *t*, *r*) with confidence intervals, effect sizes, degrees of freedom and *P* value noted<br>*Give P values as exact values whenever suitable.*
☒	☐	For Bayesian analysis, information on the choice of priors and Markov chain Monte Carlo settings
☒	☐	For hierarchical and complex designs, identification of the appropriate level for tests and full reporting of outcomes
☒	☐	Estimates of effect sizes (e.g. Cohen's *d*, Pearson's *r*), indicating how they were calculated

Our web collection on statistics for biologists contains articles on many of the points above.

Software and code

Policy information about availability of computer code

Data collection	Fiber photometry data was collected with Thinker TechFiber Photometry. Behavioral experiments (EPM, OFT, TST and social interaction) were assayed using video tracking software Ethovision XT 8.5 (Noldus). Behavioral data (fear conditioning) was collected with Med-PC software suite (Med Associates, Inc.). Microscopy data was collected with Nikon A1 Application Suite software (NIS-AR V5.02).Quantative PCR data was collected with Eppendorf realplex2 Mastercycler ep realplex software.
Data analysis	MATLAB R2019a:Custom fiber photometry data analysis. Clever System software: EPM, OFT, TST and social interaction. Med-PC software suite: fear conditioning. Image J: Western blot lane gray analysis. Graphpad Prism version 8 and SigmaPlot 12.5: All Statistical analysis. Graphpad Prism version 8: Plotting. Adobe illustrator CS6: Figure typography.

For manuscripts utilizing custom algorithms or software that are central to the research but not yet described in published literature, software must be made available to editors and reviewers. We strongly encourage code deposition in a community repository (e.g. GitHub). See the Nature Portfolio guidelines for submitting code & software for further information.

Data

Policy information about availability of data

All manuscripts must include a data availability statement. This statement should provide the following information, where applicable:

- Accession codes, unique identifiers, or web links for publicly available datasets
- A description of any restrictions on data availability
- For clinical datasets or third party data, please ensure that the statement adheres to our policy

All data that support the findings of this study are available from corresponding authors on request.

Human research participants

Policy information about studies involving human research participants and Sex and Gender in Research.

Reporting on sex and gender	NA
Population characteristics	NA
Recruitment	NA
Ethics oversight	NA

Note that full information on the approval of the study protocol must also be provided in the manuscript.

Field-specific reporting

Please select the one below that is the best fit for your research. If you are not sure, read the appropriate sections before making your selection.

☒ Life sciences ☐ Behavioural & social sciences ☐ Ecological, evolutionary & environmental sciences

For a reference copy of the document with all sections, see nature.com/documents/nr-reporting-summary-flat.pdf

Life sciences study design

All studies must disclose on these points even when the disclosure is negative.

Sample size	No statistical methods were used to predetermine sample size. Our sample sizes were estimated based on previous experience and are similar to those generally employed in the field.
Data exclusions	The animals with miss viral injection in the targeted area were excluded.
Replication	All the experiments were independently replicated with 2-3 parallel experiments.
Randomization	Mice were randomly assigned to experimental and control groups.
Blinding	The memory retention tests were taped by a digital video camera, and a trained observer blind to the genotype and treatment analysis the duration of interaction with the novel mouse with Ethovision XT 8.5 tracking system.

Reporting for specific materials, systems and methods

We require information from authors about some types of materials, experimental systems and methods used in many studies. Here, indicate whether each material, system or method listed is relevant to your study. If you are not sure if a list item applies to your research, read the appropriate section before selecting a response.

Materials & experimental systems

n/a	Involved in the study	
☐	☒	Antibodies
☒	☐	Eukaryotic cell lines
☒	☐	Palaeontology and archaeology
☐	☒	Animals and other organisms
☒	☐	Clinical data
☒	☐	Dual use research of concern

Methods

n/a	Involved in the study	
☒	☐	ChIP-seq
☒	☐	Flow cytometry
☒	☐	MRI-based neuroimaging

Antibodies

Antibodies used

Primary antibodies:
Mouse anti-TH: Sigma-Aldrich, 1:1000, Cat#AB152
Rabbit anti-RFP: Rockland, 1:500, Cat#600-401-379
Rabbit anti-GFP: Thermo Fisher Scientific, 1:500, Cat#A-10262
Rabbit anti-pERK: Cell Signaling Technology, 1:1000, Cat#9101S
Mouse anti-ERK: Cell Signaling Technology, 1:2000, Cat#4696S
Secondary antibodies:
Goat anti-rabbit, Alexa-488: Jackson Immuno Research, 1:50000, Cat#111-545-144
Goat anti-rabbit, Cy3: Jackson ImmunoResearch, 1:50000, Cat#115-165-116
Rabbit Antibody Dylight™ 800: Rockland, 1:50000, Cat#611-145-002
Mouse Antibody Dylight™ 680: Rockland, 1:50000, Cat#610-144-002

Validation

Primary antibodies:
Mouse anti-TH: PMID: 26232228
Rabbit anti-RFP: PMID: 26023136
Rabbit anti-GFP: PMID: 28386011
Rabbit anti-pERK: PMID: 12832561
Mouse anti-ERK: PMID: 34911940

Animals and other research organisms

Policy information about studies involving animals; ARRIVE guidelines recommended for reporting animal research, and Sex and Gender in Research

Laboratory animals

wild-type C57BL/6: Obtained from Slaccsas Lab, (Shanghai, China). TH-Cre mice: Obtained from Jackson Laboratory #008601, (CA,USA). Adrb1 flox and Adrb2 flox transgenic mice were obtained from Biocytogen Pharmaceuticals(Beijing, China). Arrb2 flox transgenic mice were provided by Professor Pei Gang (Shanghai Institutes for Biological Sciences, Chinese Academy of Sciences).

Wild animals

This study did not involve wild animals.

Reporting on sex

All mice used in this study were male.

Field-collected samples

This study did not involve samples collected from the field.

Ethics oversight

Experimental procedures were approved by Animal Care and Use Committee of Shanghai Medical College of Fudan University.

Note that full information on the approval of the study protocol must also be provided in the manuscript.
